# Lives of a Cell: 40 Years Later, A Third Interpretation

**DOI:** 10.3201/eid2104.110793

**Published:** 2015-04

**Authors:** Virginia M. Dato

**Affiliations:** Pennsylvania Department of Health, Pittsburgh, Pennsylvania, USA

**Keywords:** DNA, laboratories, United States Department of Agriculture, USDA, ecology, physicians, veterinarians, world health, bacteria, viruses, One Health

In 1974, Lewis Thomas (1913–1993), physician, professor, and dean, published The Lives of A Cell (*1*), the first of 2 books subtitled Notes of a Biology Watcher (*1*,*2*). The phrase “lives of a cell” refers to the independent yet interrelated parts of a human cell—including mitochondria, centrioles, and basal bodies—that once led independent lives. Without these previously independent lives working together, we would not have the capacity for thought, communication, and movement. Dr. Thomas wrote, “Our membranes hold against equilibrium, maintain imbalance, bank against entropy…. We are shared, rented and occupied.”

Our human lives do not depend just on the lives in our individual cells. Our lives depend fully on the earth, including the atmosphere, and the many other human and nonhuman lives that occupy it. In explaining this complex interdependence, Dr. Thomas observed that the earth is “most like a cell.” This second interpretation of lives of a cell refers to the many interrelated earthly entities, such as plants, whales, humans, and even viruses, that “dart rather like bees from organism to organism, from plant to insect to mammal to me and back again,” all protected by the sky—a membrane that “works, and for what it is designed to accomplish it is as infallible as anything in nature.”

PulseNet identifies disease outbreaks by connecting DNA “fingerprints.” PulseNet, established by the Centers for Disease Control and Prevention (Atlanta, GA, USA) in 1996, comprises 87 laboratories in all 50 states (*3*,*4*). The labs develop and compare DNA patterns from bacterial pathogens submitted by state, Food and Drug Administration, and US Department of Agriculture laboratories from across the nation (*5*). The work of PulseNet provides insight into the lives of a bacterial cell through DNA pattern matches. For some matches, the connection is clear; for other matches, no connection is known. Yet these bacteria are not just the same strain or type; they have identical or nearly identical patterns of DNA.

In his essay “The Wonderful Mistake” (*2*), Dr. Thomas wrote, “The capacity to blunder slightly is the real marvel of DNA. Without this special attribute, we would still be anaerobic bacteria and there would be no music.” Some DNA differences result from blunders during the DNA replication necessary for cell division. Others result from mobile genetic elements—snippets of DNA that are able to move between bacteria (*6*). Suddenly a cell exists that creates a brand new pattern. As each of the cell progeny divides, the new pattern lives on in each new clone. These progeny are the lives of a cell.

Some patterns are stable and spread so far and wide so long ago that they exist throughout nature. The PulseNet specimens have no connection other than symbiotic flora, colonization, contamination, or infection by bacteria that at some point—years ago—were progeny of the same cell. Then one of those cells mutates through a DNA insertion, deletion, or point mutation, and suddenly a new pattern appears. If the lives of this cell appear in 2 specimens sent to PulseNet, connections are revealed across time and space.

A factory produces a contaminated product, and the lives of a cell connect a retired person on a limited income to a young adult consuming the same product in a different county. A contamination in dog food can sicken a dog’s human owners, while the dogs remain healthy. Contamination on a farm can send lives of a cell across the country to rich and poor.

Dr. Thomas described a “half way technology” (*1*); “at the same time highly sophisticated and profoundly primitive.” It represents things “one must continue to do until there is a genuine understanding of the mechanisms involved in disease.” Examples included organ transplants and iron lungs. Like these, connecting lives of a cell can be labor- and resource-intensive.

In the case of human infections, unavoidable delays occur between the time a person is exposed to bacteria, becomes sick, and provides a sample for culture; the culture grows; the bacterium is identified in the laboratory, grown on media for transportation, and sent to a second laboratory; a pattern is determined; and matches are identified. After a match is determined, investigations often require repeated phone calls, followed by intensive testing of products and finally massive recalls. If the pattern disappears, investigations may be suspended before a common source is found.

All 3 interpretations of lives of a cell—the once independent lives of a single cell, the many lives (human, animal, bacterium, fungus, virus) of the earth, and the lives of a bacterial cell that travels throughout the earth—strongly suggest a need for multidisciplinary and interdisciplinary collaborations, i.e., “One Health.” The One Health Initiative—One World, One Medicine, One Health—has as its foundation the work of Dr. Thomas and many other great scientists. The One Health vision is to improve (and help save) “the lives of all species—human and animal—through the integration of human medicine, veterinary medicine and environmental science” (http://www.onehealthinitiative.com/mission.php).

Publicity about recalls may leave the perception that our food supply is riskier than before PulseNet. On the contrary, following the lives of a cell leads to a new understanding of disease mechanisms. As many diverse professionals work together and pool knowledge to develop economical solutions, our food supply becomes safer. This is the “real high technology” that Lewis Thomas described in The Technology of Medicine (*7*). “When it becomes available it is relatively inexpensive, simple, efficacious, expeditious and easy to deliver”—and is thus One Health in action (*8*).
